# The effect of Maras powder and smoking on the microRNA deregulation of oral mucosa

**DOI:** 10.1590/1678-7757-2019-0382

**Published:** 2020-01-31

**Authors:** Betül TAŞ, Ali Osmay GÜRE

**Affiliations:** 1 Gaziantep University Faculty of Dentistry Department of Oral and Maxillofacial Surgery Gaziantep Turkey Gaziantep University, Faculty of Dentistry, Department of Oral and Maxillofacial Surgery, Gaziantep, Turkey.; 2 Bilkent University Department of Molecular Biology and Genetics Ankara Turkey Bilkent University, Department of Molecular Biology and Genetics, Ankara, Turkey.

**Keywords:** MicroRNAs, Smokeless tobacco, Smoking, Mouth mucosa

## Abstract

**Objective:**

This study aimed to investigate the effects of Maras powder (a type of smokeless tobacco obtained from *Nicotiana rustica* Linn and mixed with the ashes of wood, especially from oak, walnut or grapevine) on the microRNA (miRNA) deregulation of oral mucosa, and it compares these effects with those of smoking.

**Methodology:**

Oral mucosal samples were collected from 74 patients, consisting of 16 nonusers, 26 smokers, and 32 Maras powder users. Genes associated with oral cancer were selected and 90 microRNAs targeting these genes were identified. MicroRNA were isolated and purified using the microRNA isolation kit. MicroRNA were expressed using Fluidigm RT-PCR.

**Results:**

A positive correlation between the duration of Maras powder use with miR-31 expression levels, and a negative correlation between the Maras powder chewing time and miR-372 expression levels was found. In addition, there is a negative correlation between the amount of Maras powder consumed and expression levels of miR-375, miR-378a, miR-145, and miR-10b; moreover, another negative correlation is observed between the number of cigarettes consumed and the expression levels of miR-23a, miR-23b, miR-203a, miR-200b, and miR-375. However, miR-200b and miR-92a levels were downregulated significantly more in Maras powder users when compared with smokers and nonusers (p<0.05).

**Conclusion:**

The results show both chewing Maras powder and smoking have an effect on deregulation of miR-200b and miR-92a expressions. This leads to the belief that assessing the expression of these two miRNAs is a promising noninvasive method of analysis, especially in mutagen exposures. Finally, large-scale and high-throughput studies may help to identify an extensive miRNA expression profile associated with tobacco use and improve the understanding of oral malignancies.

## Introduction

MicroRNAs (miRNAs) are endogenous small noncoding RNAs that function in messenger RNA (mRNA) silencing and in the post-transcriptional regulation of gene expression^1^ . MiRNAs are important in various cellular processes, such as proliferation, differentiation, cell growth, and cell death^2^ . Recent studies have discovered deregulated expression of miRNAs in oral infections, periodontal diseases, and oral cancer^1 , 3 , 4^ . Xie, et al.^1^ (2011) performed a preliminary comparison of healthy and inflamed gingiva (10 healthy and 10 inflamed gingiva). They found 12 miRNAs, functioning in inflammatory processes and expressed differently, and reported a probable close relationship between miRNAs and periodontal diseases^1^ . Similarly, Nahid, et al.^5^ (2011) found a persistent association between periodontal pathogens and miR-146a expression, suggesting miRNAs may promote periodontal diseases^5^ . Likewise, understanding the relationship between miRNAs and oral malignancies has become increasingly important. Park, et al.^6^ (2009) evaluated and compared the miRNA expression profiles of oral squamous cell carcinoma patients and healthy controls, and this group identified two differentially expressed miRNAs (miR-200a and miR-125a) in carcinoma patients. In addition to the oral cancer, oral precancerous lesions also affect deregulation of miRNA expression^4^ .

Tobacco products vary in the way they are consumed, for example, as cigarettes, cigars and cigarillos, which are smoked; also as smokeless tobaccos, such as chewing tobacco, snuffs, and dissolvable products. Yet, tobacco is mainly consumed in the form of manufactured cigarettes. According to the Global Adult Tobacco Survey conducted in 2015, smoking prevalence was 27.1% where smokeless tobacco use was not included^6^ . However, Akbay and Kafas^7^ (2017) reported that 16.7% of all participants were chewing MP, while 36.8% of all tobacco usage in South-East Turkey is of smokeless tobacco. The smokeless tobacco used in Turkey comes from *Nicotiana rustica* Linn and is called Maras powder (MP). To prepare for consumption, it is mixed with the ashes of wood – especially oak, walnut, or grapevine –, in approximate ratios of 1:2 or 1:3. The ash helps the absorption via oral mucosa by creating an alkaline environment^8^ . The mixture is placed between the lips or the cheeks and gums/teeth and kept in the mouth for 5-10 minutes.

Despite the hazardous effects of tobacco on health, molecular alterations in the oral mucosa of tobacco chewers and smokers have not been fully investigated^9^ . Experimental methods revealed miRNA expression in oral fibroblasts and oral keratinocytes is deregulated by the exposure to tobacco condensate^9^ .

Because of this knowledge, this study hypothesized that smokeless tobacco use and smoking can deregulate miRNA expression profiles. Therefore, it aimed to analyze the expression profiles of 90 miRNAs (including miR-92a, miR-200b, miR-31, miR-372, miR-375, miR-378a, miR-145 and miR-10b, miR-23a, miR-23b, miR-203a, and miR-375) in a cross-sectional study to understand how chronic exposure of oral mucosa to tobacco affects the miRNA expression.

## Methodology

This study was conducted at the Oral and Maxillofacial Surgery Department of the Faculty of Dentistry at Gaziantep University. Seventy-four male participants (aged between 21 and 54 years), consisting of 16 nonusers (no history of tobacco use), 26 smokers and 32 MP users, were included in this study. It was confirmed that smokers had never used MP and that MP users had never smoked.

This study followed the medical protocol set out by the Declaration of Helsinki and received ethical approval from Gaziantep University, Clinical Research Ethics Committee (2014/431). All participants signed a consent form stating their agreement to participate in the study. Data pertaining to these individuals were collected using a standardized questionnaire, which investigated age, sex, and smoking/chewing habits.

### Inclusion and exclusion criteria

Volunteers between 18 and 65 years of age with no history of disease and acute/chronic inflammation were selected. Those who had been receiving a treatment regimen for less than 6 months were excluded. Those with presence or history of potentially premalignant oral lesions or oral cancer, dermatological diseases, oral infections, fractured jaw or previous surgery that could affect oral health, and those that consumed alcohol, narcotics, or tobacco products other than MP or cigarettes were also excluded.

### Sample collection

Oral samples were collected by scraping oral mucosa in one direction using a brush or surgical blade. The samples were stored at −80°C in empty specimen containers until being anayzed^1^ . Samples were collected from the buccal mucosa for smokers and nonusers, and from the application site for MP users.

### RNA isolation and cDNA synthesis

Genes associated with oral cancer were selected based on the literature, and 90 miRNAs targeting these genes were identified using the TargetScan and miRDB databases. Furthermore, RNU6, Hs_SNORD68_11 and miRTC control, known to have relatively stable expression levels across different cell and tissue types^10^ , were used as an internal reference for normalization.

MiRNAs were isolated using the miScript primer assay kit (Qiagen, Santa Clarita, CA, USA), according to the manufacturer’s instructions. Isolated miRNAs were reverse transcribed to generate complementary DNAs (cDNAs) with the miScript II RT kit (Qiagen, Santa Clarita, CA, USA). To increase the amount of cDNA, a pre-amplification step was performed (miScript PreAMP PCR kit – Qiagen, Santa Clarita, CA, USA). cDNA obtained after reverse transcription was stored at -20°C until its use.

Real-time PCR was performed using a Rotor Gene 6000 Real-Time PCR Machine with the miScript SYBR Green PCR Kit (Qiagen, Santa Clarita, CA, USA) for miRNA expression.

### Bioinformatics and statistical analysis

Threshold cycle (Ct) values were obtained for the miRNAs and normalized using the internal control genes, respectively. All Ct analyses were performed using R Programming Language v3.2.2. Four miRNAs (hsa-miR-762, hsa-miR-371-5p, hsa-miR-4685-5p and hsa-miR-181d-3p) were excluded from the analyses for not having adequate Ct values. The expression profiles of 86 miRNAs were then analyzed. Due to the exponential nature of PCR data, data was transformed to log base 2 to achieve a normal distribution. The relative differences in expression (miRNA expression about the internal controls) were determined using the comparative threshold cycle (delta-delta Ct) method. Fold change calculations were based on the formula 2^CT^. The mean values of the control genes (RNU6, Hs_SNORD68_11, and miRTC control) were obtained to calculate 2^CT^.

Statistical analysis was performed using SPSS Version 22.0 (IBM, NY, USA). Variables were described using standard deviation (SD), standard error (SE), the range (minimum-maximum), and a 95% confidence interval for the mean. The One-way Anova test (F) was used to examine the differences between the miRNA expression profiles of the three groups. It was then followed by Duncan’s *post-hoc* test for binary comparisons. The duration of usage, the amounts of MP or numbers of cigarettes consumed, and the amount of time that MP is chewed *per* day and the area where it is applied were analyzed using the Pearson’s correlation test. p<0.05 was considered statistically significant.

## Results

**Figure 1:**
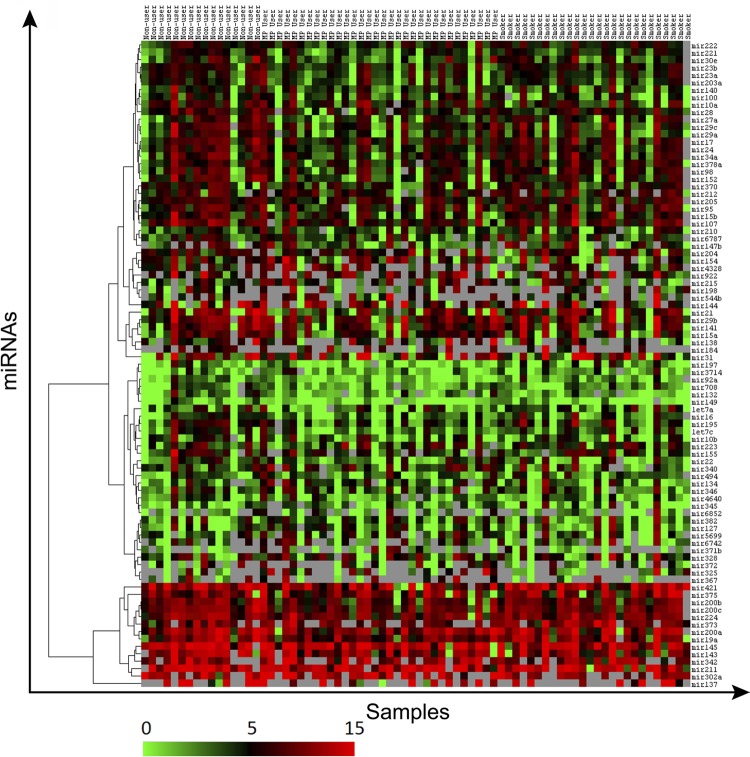
A heat map representing microRNA expression in nonusers, MP users and smokers. The clustering tree showing the concordance between miRNAs shown in the Y axis. Red tones on the heat map indicate higher expression levels than the median, while green tones show lower expression levels than the median

All individuals who participated in this study were men. The mean age of the individuals was 32.02 (±8.13) and all study groups were age-homogeneous (f=1.371, p>0.05). The intention was to investigate the expression profiles of 90 miRNAs; however, four miRNAs (miR-762, miR-371-5p, miR-4685-5p, and miR-181d-3p) were excluded from the study for not having adequate Ct values. The expression profiles of the 86 remaining miRNAs were analyzed ( Figure 1 ).

**Figure 2:**
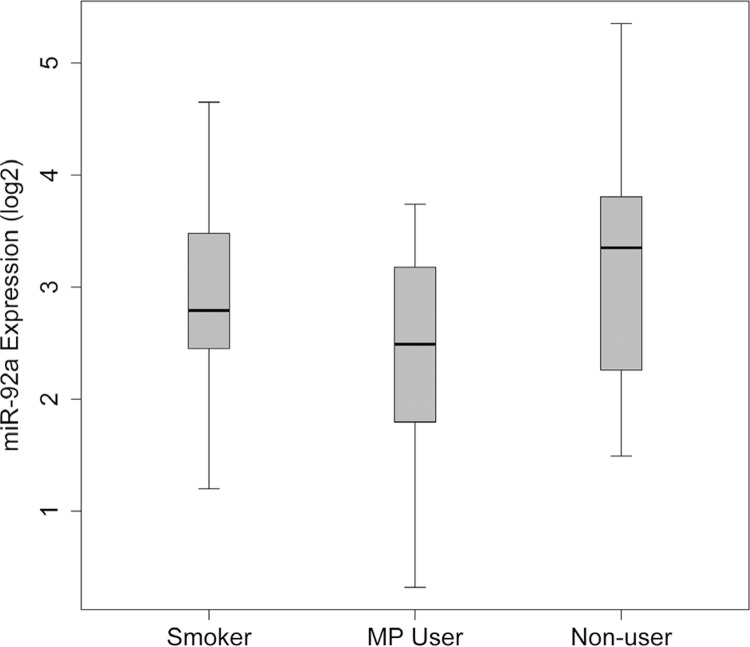
Boxplot of One-way Anova results for miR-92a expression

Of the 86 miRNAs, only miR-92a and miR-200b showed different expressions between MP users, smokers, and nonusers. The mean (±SD) expression level of miR-92a was 5.979 (±1.22) in smokers, 5.343 (±1.62) in MP users, and 6.461 (±1.22) in nonusers ( Figure 2 ), and the difference was statistically significant between the groups (f=4.790, p=0.011). *Post-hoc* analysis identified no significant difference between smokers and nonusers for miR-92a expression (p>0.05), while that of MP users was significantly different from that of smokers and nonusers (p<0.05) ( Table 1 ).

**Table 1:** Differentially expressed miRNAs related to duration and consumption of MP and smoking

miRNA		N	Mean	SD	F	p
miR-200b	Smoker	25*	2.998	0.958	3.606	0.032
	MP	32	2.344	0.950		
	Nonuser	16	3.127	1.042		
miR-92a	Smoker	25*	5.979	1.229	4.790	0.011
	MP	32	5.343	1.620		
	Nonuser	16	6.461	1.229		

*Results of one sample were missing for related miRNAs

**Figure 3:**
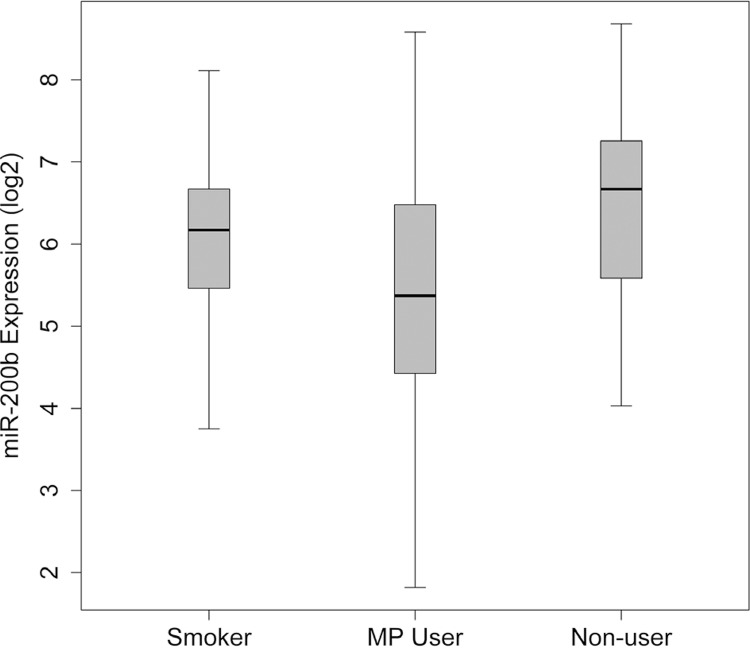
Boxplot of One-way Anova results for miR-200b expression

The mean (±SD) expression level of miR-200b was 2.998 (±0.95) in smokers, 2.344 (±0.95) in MP users, and 3.127 (±1.04) in nonusers ( Figure 3 ). The difference in miR-200b expression was statistically significant between the groups (f=3.606, p=0.032). *Post-hoc* analysis identified miR-200b significantly altered in MP users compared with nonusers (p<0.05).

The mean duration of MP use was 9.68 (±5.59) years, while the mean consumption amount was 0.64 (±0.46) packets *per* day. In addition, MP is chewed for 81.40 (±43.79) minutes *per* day. The mean duration of smoking was 13.34 (±8.02) years, while the mean consumption amount was 1.00 (±0.32) packet *per* day.

It was determined that the duration of MP use was positively correlated with miR-31 expression (r=0.444, p=0.016) and the MP chewing time *per* day was negatively correlated with miR-372 (r=−0.628, p=0.005).

While the consumption amount of MP was negatively correlated with miR-375 (r=−0.354, p=0.047), miR-378a (r=−0.390, p=0.03), miR-145 (r=−0.356, p=0.046) and miR-10b (r=−0.357, p=0.045) expression, it was positively correlated with miR-138 expression (r=0.484, p=0.03).

The amount of cigarettes consumed was negatively correlated with miR-23a (r=−0.428, p=0.033), miR-23b (r=−0.441, p=0.027), miR-203a (r=−0.522, p=0.008), miR-200b (r=−0.410, p=0.042), and miR-375 expression (r=−0.475, p=0.016).

## Discussion

Tobacco use (via smoking cigarettes or other forms of exposure to tobacco constituents) is closely associated with cardiovascular and respiratory diseases. It is also the most important risk factor for cancer and is responsible for the increased risk of death from all causes^11^ . Besides, tobacco use affects inflammatory processes and causes dysplastic changes in oral tissues by changing the apoptotic function. A dysfunctional apoptotic system can contribute to the pathogenesis of many diseases, including oral pathologies^10^ . MP causes genotoxic changes in oral mucosa^10 , 12^ . An epidemiological study revealed that 9% of 80 individuals using MP had dysplasia, carcinoma *in situ* , and cancer correlated with the duration of consumption^13^ .

MP consumption is rare among women in Turkey. Therefore, only male subjects were included in our study. Concerning this topic, Akbay and Kafas^8^ (2017) reported that among participants using MP, 1.4% were women and 25.1% were men.

Tobacco products modulate the turnover of epithelial and mesenchymal tissues^14^ and inhibit cell apoptosis, playing a significant role in oral carcinogenesis^15^ . Bhat, et al.^10^ (2018) reported that tobacco use alters miRNA expression in oral cells. This leads us to investigate the miRNA expression of oral mucosa in two frequently used forms of tobacco. To the best of our knowledge, we are the first group to investigate the effect of MP use on miRNA expression. From the 90 miRNAs analyzed, only miR-92a and miR-200b showed different expressions between MP users, smokers, and nonusers. The downregulation of the miR-200 family is allegedly a sign of inhibitory effects on cell proliferation, invasion, and metastasis and functions as a tumor suppressor^16^ . In our study, the mean expression level of miR-200b was lower in MP users than in smokers and in nonusers, and a significant difference was observed between MP users and nonusers. Similar to our findings, Bhat, et al.^10^ (2018) determined that miR-200b found to be downregulated in oral keratinocytes exposed to cigarette smoke. MiR-92a is suggested to serve as an oncogene or tumor suppressor in different cancers. In this study, the mean expression level of miR-92a was lower in MP users than in smokers and in nonusers, and there was a significant difference between MP users and smokers, and between MP users and nonusers.

Our results showed miR-31 is positively correlated with MP use, similar to those of Bhat, et al.^10^ (2018), who found upregulation of miR-31 in smokeless tobacco-treated oral cells. MiR-31 is also upregulated in oral premalignant epithelium and epithelial dysplasia and in several neoplasms, including head and neck cancers^17^ .

The number of cigarettes consumed was negatively correlated with miR-23a, miR-23b, miR-203a, miR-200b, and miR-375 expression. Many studies have been conducted to evaluate the effect of smoking on miRNA regulation. Smoking has been reported to reduce miR-203a expression in rectal cancer^18^ . Also, Ma, et al.^19^ (2014) stated that the dysregulation of miR-23a and miR-23b may be implicated in the progression of human gastric cancer, and the combined expression of miR-23a and miR-23b seems to be a valuable marker for prognosis of this disease^19^ .

This study, interestingly, determined that both the amount of MP and number of cigarettes consumed were negatively correlated with miR-375. Similarly, Conickx, et al.^14^ (2017) showed the expression of miR-375 decreased significantly in the lungs of mice after exposure to cigarette smoke for 24 weeks^13^ .

MiRNA profiling was performed on oral mucosa, although blood could be tested to determine the correlation between tissue and blood. In addition, histological analysis could be conducted to support our findings. The sample size is our limitation in this study, as MP is usually consumed with cigarettes and/or is used as an alternative to smoking. Multicenter longitudinal studies investigating larger sample sizes are needed to generalize our findings. In future studies, examining the changes in existing miRNA expressions after the cessation of tobacco use will also be necessary.

## Conclusions

The results of our study show the use of MP and smoking deregulate miRNA expression (miR-200b and miR-92a). This leads to the belief that assessing miRNA expression is a promising noninvasive method of analysis, especially in the case of mutagen exposures. Finally, large-scale and high-throughput studies may help to identify extensive miRNA expression profiles associated with tobacco use and improve the understanding of oral malignancies.

### Ethical approval

This study was approved by Gaziantep University Clinical Research Ethics Committee. All procedures involving human participants were conducted according to the ethical standards of the institutional and/or national research committee and with the 1964 Declaration of Helsinki and its later amendments or comparable ethical standards.
